# Recurrent Metastasized Parathyroid Carcinoma—Long‐Term Remission After Combined Treatments With Surgery, Radiotherapy, Cinacalcet, Zoledronic Acid, and Temozolomide

**DOI:** 10.1002/jbm4.10114

**Published:** 2018-11-29

**Authors:** Sara Storvall, Eeva Ryhänen, Frank V Bensch, Ilkka Heiskanen, Soili Kytölä, Tapani Ebeling, Siru Mäkelä, Camilla Schalin‐Jäntti

**Affiliations:** ^1^ Endocrinology, Abdominal Center University of Helsinki and Helsinki University Hospital Helsinki Finland; ^2^ Radiology University of Helsinki and Helsinki University Hospital Helsinki Finland; ^3^ Endocrine Surgery Abdominal Center University of Helsinki and Helsinki University Hospital Helsinki Finland; ^4^ Laboratory of Genetics, HUSLAB Helsinki University Hospital Helsinki Finland; ^5^ Endocrinology University of Oulu and Oulu University Hospital Oulu Finland; ^6^ Department of Oncology Comprehensive Cancer Centre University of Helsinki and Helsinki University Hospital Helsinki Finland

**Keywords:** HYPERCALCEMIC CRISIS, TREATMENT, TEMOZOLOMIDE, MGMT METHYLATION

## Abstract

Parathyroid carcinoma is a rare cause of primary hyperparathyroidism with rather poor prognosis. Apart from surgery, no evidence‐based treatments exist. A 48‐year‐old woman presented with weight loss, nausea, constipation, hypercalcemic crisis, and a recurrent neck tumor 5 years after primary surgery of a parathyroid tumor that primarily was classified as an adenoma. Histopathological reevaluation of the original tumor revealed the correct diagnosis to be parathyroid carcinoma (PC). The patient underwent surgery of the recurrent tumor, which was locally invasive with metastatic spread to the mediastinum and neck lymph nodes. Computed tomography demonstrated large lytic bone lesions in both iliac bones including, on the right, a soft tissue mass compatible with bone metastasis. The patient was treated with cinacalcet, repeated zoledronic acid infusions, and temozolomide cycles for 1 year. She underwent two additional neck surgeries for PC and sternotomy for resection of mediastinal metastases. Massive osteolytic lesions in both femoral necks caused imminent fracture risk and therefore both femurs were prophylactically stabilized by intramedullary nail. Serum calcium normalized after the third neck surgery, cinacalcet was discontinued, and parathyroid hormone gradually normalized during continued treatments with temozolomide, adjuvant radiotherapy, and zoledronic acid, with no signs of active disease on imaging and normal biochemistry. The patient remains in remission 17 years after successful combined treatments for recurrent, metastasized PC. The parathyroid tumor tissue demonstrated high O^6^‐methylguanine DNA methyltransferase (MGMT) promoter methylation status, a known predictor of positive temozolomide treatment response in other tumors. In addition, synergistic effects of multiple treatments may have accounted for the favorable response. © 2018 The Authors. *JBMR Plus* is published by Wiley Periodicals, Inc. on behalf of the American Society for Bone and Mineral Research.

## Introduction

Parathyroid carcinoma (PC) accounts for approximately 1% of primary hyperparathyroidism and is an extremely rare endocrine cancer.^1^, ^2^, ^3^ Recent reports indicate increasing incidences in the United States, Australia, and Europe.^1^, ^4^, ^5^ PC can occur sporadically or as part of hereditary syndromes such as multiple endocrine neoplasia (MEN), familial isolated primary hyperparathyroidism, and/or hyperparathyroidism jaw‐tumor syndrome (HPT‐JT). Germline and/or somatic mutations in the *CDC73* suppressor gene underlie familial isolated hyperparathyroidism and HPT‐JT.^1^, ^2^, ^3^, ^6^, ^7^ PC tends to progress aggressively with recurrence rates of 40% to 60% and median overall survival of 14.3 years.^1^, ^8^, ^9^ The only known curative treatment is surgery. Inoperable/disseminated PC carries a high mortality rate of 35%.^8^ Patients often succumb to uncontrollable hypercalcemia and renal insufficiency rather than to the tumor burden itself.^10^ We present a now 64‐year‐old woman with recurrent PC that had metastasized to the mediastinum, with lytic bone lesions and possible metastases in the surrounding soft tissues, who was treated with a combination of surgery, radiotherapy, cinacalcet, repeated zoledronic acid infusions, and temozolomide (TMZ) cycles with resulting excellent treatment response.

## Materials and Methods

All biochemical measurements were performed at the Helsinki University Central Hospital Laboratory (HUSLAB) using in‐house methods. MGMT promoter methylation status was determined from previously isolated tumor DNA. By sodium bisulfite treatment of DNA (EpiTect Bisulfite Kit, Qiagen, Finland) unmethylated cytosine residues were converted to uracils while methylated residues were left unchanged. After PCR, the methylation of the five CpGs and one control CpG was analyzed by a cyclic minisequencing.^11^ A mean methylation level of all investigated CpGs above 20% was classified as methylated.

Mutation studies of the *CDC73* gene were performed as previously described.^1^

## Case Report

The patient initially presented with nausea, constipation, increased thirst, weight loss, fatigue, and a palpable tumor on the right side of the neck in 2001 at 48 years of age. Fine‐needle aspiration of the neck tumor demonstrated atypical cells thought to be of thyroid origin. The patient underwent total thyroidectomy including resection of the tumor, which was adherent to the right lower part of the thyroid. Histopathological examination of the thyroid only revealed a 0.7 cm papillary microcarcinoma. The resected tumor weighed 3 g and was diagnosed as parathyroid adenoma. No follow‐up was arranged after primary surgery.

**Figure 1:**
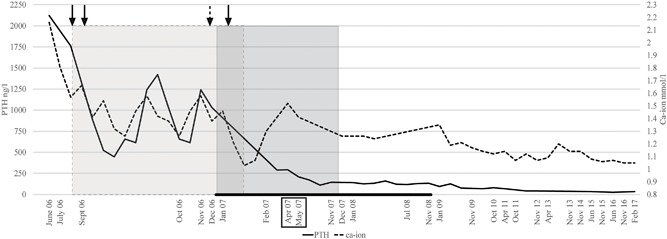
Timeline showing plasma PTH and S‐Ca‐ion concentrations. The patient was treated with cinacalcet during the time period marked as a grey area with dashed outline. Surgeries are marked with black arrows at the top, and the dashed arrow marks hip nailing. The darker gray area with continuous outline indicates the time period during which the patient was treated with TMZ. The black bar on the bottom of the chart indicates the time period during which the patient received treatment with zoledronic acid (once a month). The black box annotates radiation therapy. Note that the time intervals between points on the *x* axis are not equal.

In 2006, 5 years later, the patient was diagnosed with hypercalcemic crisis due to severe primary hyperparathyroidism (PHPT). Serum ionized calcium (S‐Ca‐ion) was 2.16 (1.16–1.30 mmol/L), fasting plasma parathyroid hormone (PTH) 2120 (15–70 ng/L) nmol/L, alkaline phosphatase (ALP) 382 U/L (35–105 U/L), and creatinine 163 μmol/L (50–90 μmol/L). Before being diagnosed with PHPT, the patient had suffered from nausea and constipation for more than 1 year and had lost 10 kg of body weight. Her general practitioner had therefore referred her for abdominal ultrasound, gastroscopy, and colonoscopy, all of which were unremarkable. She also had severe pains in her knees and shins. After emergency treatment with intravenous fluid, furosemide, and 4 mg zoledronic acid, S‐Ca‐ion decreased to 1.40 mmol/L. Fig. 1 demonstrates S‐Ca‐ion and PTH concentrations in relation to applied treatments. Ultrasound demonstrated several cystic lesions in both the right and left side of the neck. ^99m^Technetium sestamibi‐^123^iodine scintigraphy did not show any uptake in the neck. Fine‐needle aspiration of one of the lesions showed atypical cells and raised suspicion for malignancy. The patient underwent bilateral surgical neck exploration (Fig. 1) with macroscopically radical resection of a tumor in the right neck extending into the mediastinum, with invasive growth into the walls of the right common carotid artery and trachea. Histopathological examination confirmed PC with vascular infiltration. PTH stain was strongly positive, Ki‐67 proliferation index 10%, and parafibromin stain negative. Histopathological reevaluation of the neck tumor resected in 2001 also demonstrated PC.

**Figure 2:**
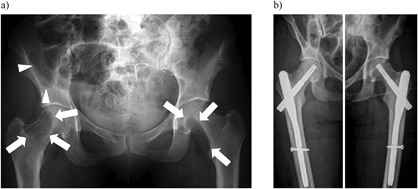
(*A*) Pelvic X‐ray with expansive lytic lesions in both femoral necks (arrows) causing an imminent threat of pathological fracture. Additionally, there is a large lytic lesion in the right iliac bone (arrowheads). November 30, 2006. (*B*) Prophylactic stabilization of the lytic lesions in both femoral necks by gamma nail for prevention of imminent pathological fracture. December 28, 2006.

**Figure 3:**
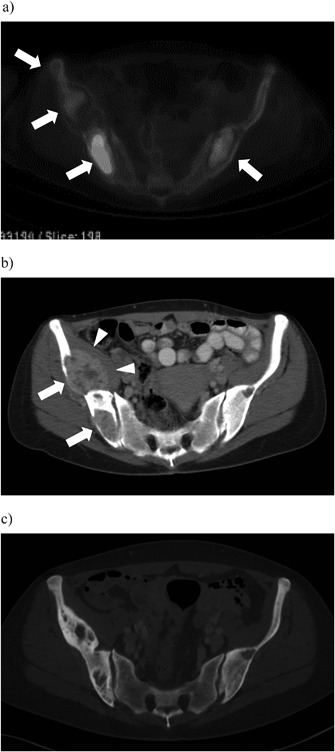
(*A*) ^18F^FDG PET/CT shows marked uptake in the lytic lesions of both iliac bones indicating high metabolic activity (arrows). January 9, 2007. (*B*) Contrast‐enhanced CT demonstrates lytic lesions within both iliac bones (arrows) with a large metastatic soft tissue mass (arrowheads) dislocating the iliacus muscle. November 30, 2006. (*C*) Contrast‐enhanced CT 10 years after successful treatment shows ossification of the old lytic lesions with no signs of aggressive expansion. March 9, 2017.

The patient was transiently normocalcemic immediately after neck surgery, but PTH concentrations remained elevated (1728 ng/L; Fig. 1). She then again developed hypercalcemia (S‐Ca‐ion 1.62 mmol/L) and treatment with cinacalcet 30 mg twice daily was initiated in August 2006. Contrast‐enhanced CT did not reveal any tumors in the neck but demonstrated a retrosternal mediastinal mass. She underwent sternotomy, her third surgery in September 2006, with resection of a tumoral mass that included the thymus. Histopathology demonstrated two PC metastases of 1.5 cm and 3 cm. Hypercalcemia persisted nonetheless, and 1 month later S‐Ca‐ion was 1.46 mmol/L, PTH 613 ng/L, and ALP 476 U/L. Pelvic X‐rays (Fig. 2 *A*) demonstrated large lytic lesions in both femoral necks and in the right iliac bone. There was intense uptake on ^18F^FDG PET/CT in both iliac bones (Fig. 3 *A*) and the surrounding soft tissues. Contrast‐enhanced CT performed in November 2006 demonstrated lytic lesions in both iliac bones and a large metastatic soft tissue mass (Fig. 3 *B*) and neck CT pathological lymph nodes in the neck and the left clavicular groove and a lytic lesion in the left clavicle. Both femurs were prophylactically stabilized by intramedullary nail in December 2006 because of imminent fracture risk. In December 2006, S‐Ca‐ion was 1.46 mmol/L, PTH 1033 ng/L, and ALP 345 U/L, and treatment cycles with TMZ were initiated; 240 mg (150 mg/m^2^) for 5 days with 28‐day intervals. There are no established systemic treatments for metastasized PC. Beneficial response to dacarbazine, an intravenously administered alkylating agent, has been reported in a few cases.^12^ TMZ, an oral alkylating agent with few side effects, also used in the treatment of other neuroendocrine tumors such as pancreatic neuroendocrine tumors and aggressive pituitary adenomas and carcinomas,^13^, ^14^ was chosen as systemic therapy. TMZ was given for 1 year (10 cycles) and was discontinued in November 2007 (Fig. 1) because the patient remained in biochemical remission and there were no signs of active disease on CT.

The patient underwent her third neck surgery in January 2007 with resection of recurrent tumors from both the left and right side of the neck (lesion size 1×3 cm and 1×2 cm, respectively). Histopathology again confirmed PC. After surgery, her S‐Ca‐ion decreased to 1.05 mmol/L and cinacalcet was discontinued (Fig. 1). In April 2007, CT imaging was normal in the neck and demonstrated decrease in size and sclerosis of the lytic bone lesions. In April 2007, she was treated with adjuvant external radiation therapy (IMRT) to the neck and upper mediastinum with a total dose of 60 Gy (Fig. 1). Zoledronic acid (4 mg iv) was continued for 10 months, according to standard guidelines on treatment of bone metastases originating from solid tumors.^15^ The patient was given altogether 11 infusions of zoledronic acid after cessation of TMZ treatment.

### Screening for possible germline mutations and determinations of MGMT promoter methylation status

The patient underwent screening for a possible underlying *CDC73* germline mutation or deletion, but the results were normal. Notably, her 33‐year‐old daughter was diagnosed with severe PHPT due to an atypical parathyroid adenoma (S‐Ca‐ion 1.96 mmol/L, PTH 823 ng/L) during pregnancy and underwent a cesarean section during the second trimester. MEN mutation screening was also negative. The patient also has a son, who is not affected by PHPT. So far, no underlying genetic defect has been identified in this family. Because the patient had a favorable response to TMZ, O^6^‐methylguanine DNA methyltransferase (MGMT) promoter methylation status was determined and demonstrated a high promoter methylation status of 35%.

In March 2017, 16 years after our patient's first surgery for PC, contrast‐enhanced whole‐body CT was unremarkable with no signs of recurrent disease and complete ossification of the old skeletal lesions (Fig. 3 *C*). The patient remains in biochemical remission (S‐Ca‐ion 1.05 mmol/L, PTH 31 ng/L, creatinine 87 µmol/L, and 25OHD 86 nmol/L) 17 years after her first surgery for PC.

## Discussion

There are no known systemic treatments for PC, the prognosis of which is especially poor in patients with distant metastases. The current case underlines many of the difficulties encountered in patients with PC: initial misdiagnosis, no follow‐up, delayed diagnosis even after recurrent disease, fine‐needle aspiration that can cause tumor rupture and seeding, hypercalcemic crises, fracture risk due to massive lytic bone lesions, as well as the challenge of treating disseminated disease. The patient underwent several surgeries. Cinacalcet was administered because of sustained severe hypercalcemia and zoledronic acid because of metastatic spread to the right iliac bone. Brown tumors due to high concentrations of PTH are deceptively similar to bone metastases on CT imaging; some of the patient's skeletal tumors were biopsied and confirmed to be Brown tumors. TMZ was chosen as systemic therapy when the disease persisted despite several surgeries. After her last neck surgery, serum calcium concentration eventually decreased significantly and cinacalcet was not needed anymore. TMZ cycles were given for 1 year. Zoledronic acid was administered for another 10 months after cessation of TMZ treatment. In the present case, these combined treatments were successful, and the patient remains in remission 17 years after her first surgery for PC.

There are anecdotal reports of successful adjuvant radiotherapy for PC.^16^, ^17^, ^18^ In disseminated disease, surgical resection of metastases decreases tumor burden and may decrease hypercalcemia and cause symptomatic relief but has not unanimously been shown to lead to survival benefit.^10^ PC is characterized by decreased or absent calcium‐sensing receptor (CaSR) expression compared to parathyroid adenomas and hyperplastic glands.^19^ Decreased CaSR and parafibromin expression is related to a more aggressive disease course.^20^ In the present case, parafibromin stain was negative, but this was not due to a *CDC73* germline mutation. Somatic mutation analyses were not performed. Cinacalcet, a calcimimetic, is an allosteric modulator of the CaSR that efficiently decreases hypercalcemia in PTH‐dependent disease. CaSR can function as an inhibitor of cell proliferation in parathyroid tumors, which was demonstrated in rodents treated with cinacalcet.^21^ Interestingly, in addition to activating CaSR, calcimimetics also seem to increase CaSR expression.^22^ It is thus possible that in the present case, cinacalcet not only decreased serum calcium concentrations but also had an effect on CaSR expression and tumor proliferation. Zoledronic acid can temporarily decrease PTH‐dependent hypercalcemia and is not only used in the treatment of osteoporosis because of its anti‐bone‐resorptive effects but also for the treatment of bone metastases, usually given in 4‐week intervals.^23^, ^24^

TMZ is an alkylating chemotherapy agent, most commonly used for the treatment of tumors of the central nervous system such as gliomas as well as for aggressive pituitary tumors and pituitary carcinoma.^25^ In the literature, we found a single report of PC treated with FOLFOX and a combination of TMZ and capecitabine, but this did not lead to treatment response.^26^ Tumors with epigenetic silencing of the MGMT gene are more susceptible to TMZ.^27^ The active metabolite of TMZ, 5‐(3‐methyltriazen‐1‐yl) imidazole‐4‐carboxamide (MTIC), produces DNA lesions such as nucleotide methylation. Methylation of the O^6^ position of guanine causes mispairing of guanine to thymine instead of cytosine. In normal cells, the MGMT enzyme restores guanine by demethylation. In MGMT‐deficient cells, repeated futile base excision mismatch repair due to persisting O^6^‐methylated guanine (O^6^‐MeG) causes extensive DNA resection and apoptosis.^28^ The MGMT enzyme thus counteracts the alkylating effects of TMZ. The possible role of MGMT gene silencing in PC has not been previously studied. The MGMT promoter methylation status in the patient's tumor was determined and found to be high, compatible with a low MGMT enzyme activity, providing the basis for a positive treatment response to TMZ. In the future, determination of MGMT promoter methylation should thus be explored in larger series of PC in order to identify patients who possibly would benefit from TMZ therapy. In addition, identification of major oncogenic tumor pathways in PC is essential and can hopefully help in targeting treatments of this rare malignancy.^6^

In conclusion, for the first time to our knowledge, we report a case of metastasized PC with high tumor MGMT promoter methylation status that benefited from TMZ treatment. In addition, synergistic effects of multiple treatments administered in the present case probably accounted for the favorable response.

## Disclosures

All authors state that they have no conflicts of interest.
